# The Prognostic Value of Low Muscle Mass in Pancreatic Cancer Patients: A Systematic Review and Meta-Analysis

**DOI:** 10.3390/jcm10143033

**Published:** 2021-07-07

**Authors:** Elisa Sefora Pierobon, Lucia Moletta, Sandra Zampieri, Roberta Sartori, Alessandra Rosalba Brazzale, Gianpietro Zanchettin, Simone Serafini, Giovanni Capovilla, Michele Valmasoni, Stefano Merigliano, Cosimo Sperti

**Affiliations:** 1 Department of Surgery, Oncology and Gastroenterology, 3rd Surgical Clinic, University of Padua, Via Giustiniani 2, 35128 Padua, Italy; elisaseforapierobon@gmail.com (E.S.P.); lucia.moletta@unipd.it (L.M.); sanzamp@unipd.it (S.Z.); gianpietro.zanchettin@gmail.com (G.Z.); simone.serafini@ymail.com (S.S.); giovannicapovilla88@gmail.com (G.C.); michele.valmasoni@unipd.it (M.V.); stefano.merigliano@unipd.it (S.M.); cosimo.sperti@unipd.it (C.S.); 2 Department of Biomedical Sciences, University of Padua, Via U. Bassi 58/B, 35121 Padua, Italy; 3 Veneto Institute of Molecular Medicine (VIMM), Via Orus 2, 35129 Padua, Italy; 4 Department of Statistical Sciences, University of Padua, Via C. Battisti 241, 35121 Padua, Italy; brazzale@stat.unipd.it

**Keywords:** low muscle mass, sarcopenia, pancreatic adenocarcinoma, pancreatic cancer, pancreatic surgery, body composition

## Abstract

Low muscle mass is associated with reduced survival in patients with different cancer types. The interest in preoperative sarcopenia and pancreatic cancer has risen in the last decade as muscle mass loss seems to be associated with poorer survival, higher postoperative morbidity, and mortality. The aim of the present study was to review the literature to compare the impact of low muscle mass on the outcomes of patients undergoing surgery for pancreatic adenocarcinoma. An extensive literature review was conducted according to the 2009 Preferred Reporting Items for Systematic Reviews and Meta-Analyses (PRISMA) guidelines and 10 articles were analyzed in detail and included in the meta-analysis. Data were retrieved on 2811 patients undergoing surgery for pancreatic cancer. Meta-analysis identified that patients with low muscle mass demonstrated a significantly reduced OS when compared to patients without alterations of the muscle mass (ROM 0.86; 95% CI: 0.81–0.91, *p* < 0.001), resulting in a 14% loss for the former. Meta-analysis failed to identify an increase in the postoperative complications and length of stay of patients with low muscle mass. Our analysis confirms the role of low muscle mass in influencing oncologic outcomes in pancreatic cancer. Its role on surgical outcomes remains to be established.

## 1. Introduction

Skeletal muscle accounts for 40–50% of the total mass in healthy-weight individuals [1] and serves as a body protein reservoir [2]. It is a plastic and highly adaptive organ that can increase or decrease its size, functional capacity, and metabolism in response to different pathophysiological stimuli. Since the muscle is an endocrine and exocrine organ, its adaptations have an impact on the entire organism’s well-being and the muscle metabolic state has been proposed as a disease modifier [2,3,4].

Pathological conditions such as cancer compromise the mechanisms that regulate muscle homeostasis, resulting in severe muscle wasting, functional impairment, and altered metabolism, impacting profoundly on the health of the host and leading to cancer cachexia syndrome.

Low muscle mass (‘secondary’ or disease-related sarcopenia) [5] is part of the diagnostic criteria to define cancer cachexia in association with body weight loss and body mass index (BMI) [6], and is associated with increased treatment toxicity and reduced survival in patients with different cancer types. In addition to low muscle mass, low muscle quality characterized by fatty infiltration (myosteatosis) is a predictor of poor outcomes after resection of various malignancies including pancreatic cancer [7,8,9,10,11,12,13,14,15].

The prognosis for pancreatic cancer is generally poor, with five-year survival rates in the range of 6% to 10% [16,17]. Radical surgical resection represents the only potential cure. Over the years, advances in surgical technique and perioperative care have led to progressive improvements of outcomes after pancreatectomy for cancer. However, postoperative morbidity rates remain high; up to 40% of patients will experience complications after surgical resection [18]. Several studies have focused on investigating preoperative factors that are able to influence postoperative course and secondary sarcopenia has been proposed as a patient-related condition with potential impacts on short and long-term surgical outcomes [19]. In fact, the interest in preoperative sarcopenia and pancreatic cancer has risen in the last decade as muscle mass and adipose tissue loss seems to be associated with higher postoperative morbidity and increased mortality [8,20,21]. Moreover, among solid tumors, pancreatic cancer carries the highest prevalence of cancer cachexia and involuntary weight loss [22]. Patients with cancer are prone to metabolic modifications, such as the Warburg effect, leading to a dramatically altered nutrient utilization [19]. Furthermore, in the case of pancreatic cancer patients, malnutrition is worsened by the exocrine insufficiency that might ensue [10].

There are multiple radiological methods that have been approved to perform body composition analysis, evaluate muscle mass, and define sarcopenia such as computed tomography (CT), magnetic resonance imaging (MRI), and dual energy X-ray absorptiometry (DXA). DXA is not usually available in cancer settings, though, and it cannot discriminate visceral adipose tissue, decipher changes between tumor mass and lean muscle mass, and it has decreased precision in obese patients [23]. Computed tomography (CT) scans have been used and proposed as the gold standard to evaluate cancer-associated changes in body composition and its association with the prognosis [11,24]. Indeed, the imaging resolution of adipose, skeletal muscles, and the precision of measures of a tissue cross-sectional area of a CT scan is excellent. Moreover, it is a practical choice as CT images are routinely acquired in the standard care of cancer patients and can provide information on body composition over time without incremental cost or radiation exposure [24]. CT scan analyses quantify skeletal muscle mass and other tissues, such as adipose or connective tissue, allowing the detection of low mass and decreased muscle radiodensity due to myosteatosis. CT image analyses reveal low levels of muscle also in individuals who are overweight or obese (sarcopenic obesity) [24,25,26,27].

The aim of the present study was to review the published literature to compare the impact of low muscle mass (evaluated by CT scan) on the short and long-term outcomes in patients with pancreatic ductal adenocarcinoma (PDAC) undergoing surgery.

## 2. Materials and Methods

### 2.1. Literature Search Strategy

Eligibility criteria were established a priori. A systematic search of literature published in English from January 2010 to September 2020 was performed to identify all original articles on patients undergoing surgical resection of PDAC in which a preoperative abdominal CT scan was used to assess skeletal muscle mass. The Preferred Reporting Items for Systematic Reviews and Meta-Analyses (PRISMA) guidelines were followed [28]. The following terms were used to search through the literature (PubMed and Web of Science databases): ‘sarcopenia’, ‘analytic morphomics’, ‘body composition’, ‘muscle depletion’, ‘muscle mass’, ‘psoas area’, ‘myopenia’, ‘core muscle’, ‘lean body mass’, or ‘muscular atrophy’, and ‘pancreatic cancer’, ‘surgery’, ‘pancreatic resection’, or ‘pancreatectomy’. The “related articles” function and all citations were used to broaden the search. Three independent researchers (ESP, LM, and GZ) reviewed the relevant titles. After excluding duplicates, abstracts were reviewed and included for initial analysis if the inclusion criteria were met. Records without abstracts, case reports, review articles, opinion articles, and experimental studies were excluded. In case of disagreement, a fourth author (MV) participated in the discussion. A manual search of the reference lists in precedent reviews and eligible articles was also performed.

### 2.2. Inclusion and Exclusion Criteria

Inclusion criteria were: (1) studies reporting the assessment of body composition by CT scan in human subjects with PDAC receiving surgical treatment; (2) body composition defined as total muscle area or total psoas area/volume at the lumbar level; (3) studies reporting on the prevalence of muscle alterations and at least one of the following outcomes: postoperative mortality, postoperative complications, length of hospital stay (LOS), disease-free survival (DFS), and overall survival (OS); and (4) studies published in English.

Exclusion criteria were: (1) review articles or case series (<5 patients); (2) publications comprising of patients with either a benign or malignant disease in which the surgical and oncological outcome were not presented separately; and (3) body composition analyzed using methods other than those described in the inclusion criteria (e.g., MRI, DEXA, etc.).

### 2.3. Measured Outcomes and Data Extraction

Data were registered in digital sheets. Data regarding authors, year of publication, country of publication, study type, characteristics of populations and of their present disease, muscle mass evaluated, cut-offs’ selection, muscle loss prevalence, incidence of major complications (graded ≥2 according to Clavien–Dindo classification [29]), DFS, and OS were retrieved. When reported by the authors, data regarding sarcopenic obesity, myosteatosis prevalence, and impact on outcomes were collected.

### 2.4. Terminology and Definitions

Regarding low muscle mass, the CT scan-determined muscle parameters, cut-off values used, muscles, and vertebral level analyzed to define low muscle mass (secondary sarcopenia) in the papers considered are reported in Table 1 and discussed in the results section. Sarcopenic obesity is defined as sarcopenia accompanied by obesity (an increase in the adipose tissue) [30]. The definitions of sarcopenic obesity used in the papers considered are reported in Table 1. Myosteatosis is the skeletal muscle fat infiltration diagnosed by CT scan-determined low muscle radiodensity (radiation attenuation in Hounsfield units). The cut-off values used to define myosteatosis in the papers considered are reported in Table 1.

### 2.5. Statistical Analysis

Three meta-analyses were conducted in line with the Cochrane Collaboration guidelines on the meta-analysis of observational studies in epidemiology [42,43]. The first analysis focused on OS in months, the second on the prevalence of major complications according to the Clavien–Dindo classification (≥2), and the third on the length of hospital stay after pancreatic surgery in patients with or without muscle loss.

A fixed-effect meta-analytical model was used for OS and major complications, whereas a random-effects meta-analytical model was used for LOS. OS was retrieved from the published studies as median values and ranges and converted into means and standard deviations (SD) using appropriate statistical algorithms according to Hozo et al [44]. The analysis requires the specification of maximal and minimal survival which was extrapolated from the figures for the purpose of this study when not clearly reported in the paper. LOS was already reported in means and SDs. Major complications are reported as percentages. The effect on the endpoints were meta-analyzed either as mean difference (MD) or as ratio of means (ROM) [43]. Values of MD < 0 or ROM < 1 indicate a disadvantage in the survival for patients with low muscle mass. The opposite holds true for the prevalence of major complications and mean LOS. Cochran’s Q statistic and the I^2^ statistic were used to test between-study heterogeneity [45]. If the Q statistic was significant at the 0.5 level, the summary effect and corresponding 95% confidence interval (CI) were obtained with the Mantel–Haenszel random effects model [46]. For I2 < 50%, between-study heterogeneity was judged to be low-moderate, while for I2 ≥ 50% it was considered substantial. The point estimate of MD and ROM was considered statistically significant when *p* was <0.05. Publication bias was assessed visually using a funnel plot and the number of missing studies was estimated using the trim-and-fill method [43].

All analyses were conducted using R version 3.5.2 [47].

## 3. Results

**Figure 1:**
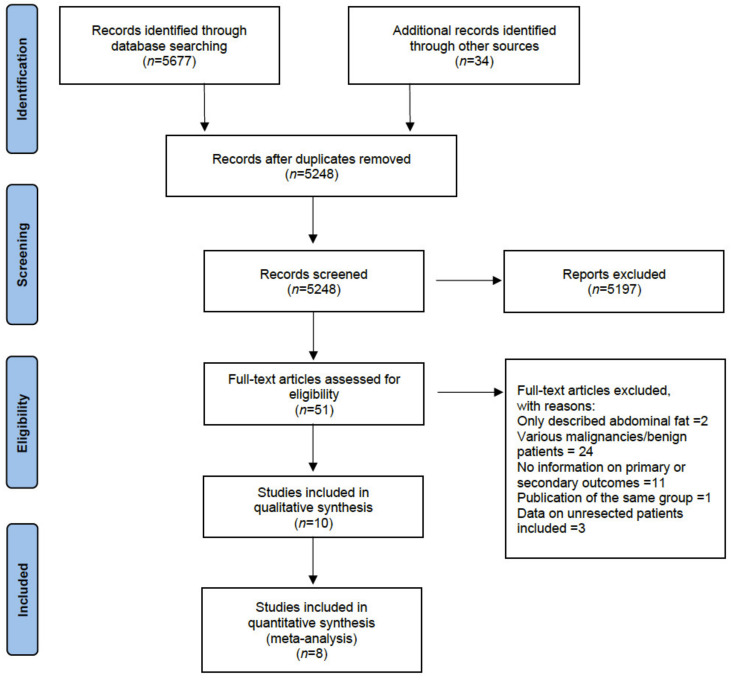
Studies’ inclusion flowchart according to the Preferred Reporting Items for Systematic Reviews and Meta-Analyses (PRISMA) guidelines [28].

The search flowchart is presented in Figure 1. A total of 5711 article titles were reviewed by following the inclusion and exclusion criteria set beforehand and after a related article and cross-reference search, a total of ten original articles in English were included in the present review. All articles were single-center retrospective cohort studies with a total of 2811 patients with PDAC undergoing surgery with curative intent. Amini et al. [32] ran two separate analyses with two different low muscle mass definitions according to the total psoas area (TPA) or total psoas volume (TPV), hence they were included individually in the meta-analysis. Studies’ characteristics are depicted in Table 2, while data used for the meta-analyses are reported in Table 3.

### 3.1. Low Muscle Mass Definitions

Six articles defined muscle mass as the area occupied by all the muscles at the level of L3 normalized for height (L3-SMI) [35,36,37,38,39,40]. Four articles defined muscle mass as the total psoas area normalized for height [31,32] or normalized for the cross-sectional area of the body at the level of L3 [34] or L5 [33]. Amini et al. also evaluated the total psoas volume normalized for height at the level of L3 (see Table 1 for details) [32]. Three articles defined low muscle mass as sarcopenia using predefined cut-offs already published in the literature [36,39,40]. Six articles [31,32,34,35,37,38] used self-determined cut-offs, whereas Delitto et al. [34] and Clark et al. [33] conducted correlation analyses as depicted in Table 1.

### 3.2. Prevalence of Low Muscle Mass in Patients with Pancreatic Adenocarcinoma

The reported prevalence of low muscle mass varies from 17.2% to 64.2% [31,32,35,36,37,38,39,40]. Two authors did not report any percentage [33,34] and one [33] did not define a cut-off as they conducted a correlation analysis to identify the relationship between low muscle mass and long-term survival. Moreover, three authors [35,38,40] reported data also regarding the prevalence of myosteatosis, ranging from 33.3% to 47.8%. Six studies [31,32,35,36,39,40] reported the numbers of sarcopenic obese patients, whose prevalence ranges from 2.5% to 25.6%.

### 3.3. Preoperative Patients’ Characteristics

Eight studies [31,32,34,35,36,38,39,40] investigated a relationship between age and low muscle mass but only 3 authors [34,36,40] found that patients with low muscle mass were significantly older. All studies reported data regarding the gender distribution of patients but only two authors [36,39] found a difference in the prevalence of low muscle mass between male and female patients with contrasting results. Specifically, higher rates of prevalence of sarcopenia were found in males by Gruber et al. [39] and in females by Ninomiya et al [36].

Six studies [34,35,37,38,39,40] reported data regarding the albumin levels and three studies [34,35,39] found significant lower levels of pre-operative albumin in the group with low muscle mass. The prevalence of diabetes was reported in two studies [38,40]. No significant difference was found in SMI values in patients with or without diabetes but sex-specific standardized skeletal muscle density was lower in diabetic patients [40]. BMI stratified according to muscle mass status was reported in five articles [35,36,38,39,40]. In four studies [35,36,38,40] BMI was significantly lower in the low muscle mass group.

Three authors [34,35,39] reported data regarding the neoadjuvant treatment. Delitto et al. reported that even if the neoadjuvant treatment was not associated with differences in the mean psoas index, a decrease in the psoas index during therapy is associated with a poor prognosis [34]. A higher rate of treated patients was found in the sarcopenic group by Gruber et al. [39] but not by Okumura et al [35].

### 3.4. Low Muscle Mass and Postoperative Outcomes

**Figure 2:**
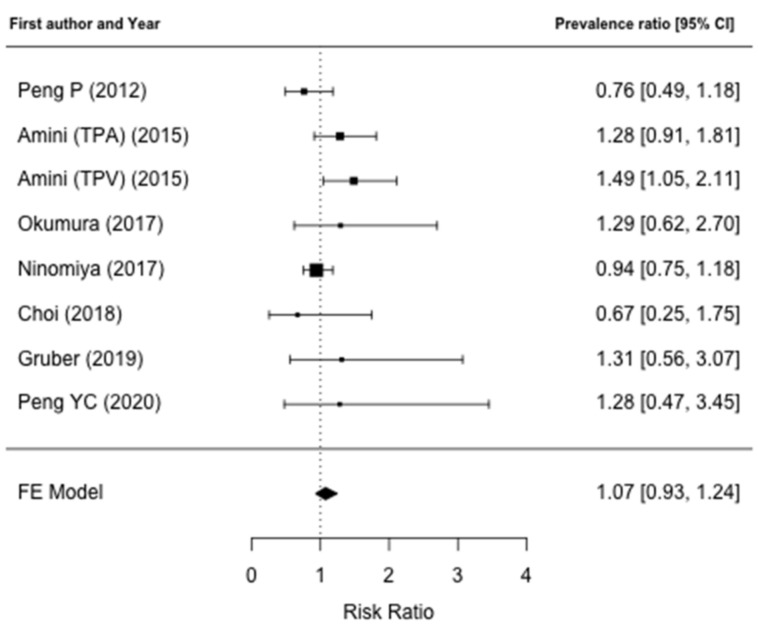
Forest plot for the prevalence ratio of major complications. Meta-analysis did not identify a higher prevalence ratio of major complications after pancreatic surgery in the low muscle mass group.

**Figure 3:**
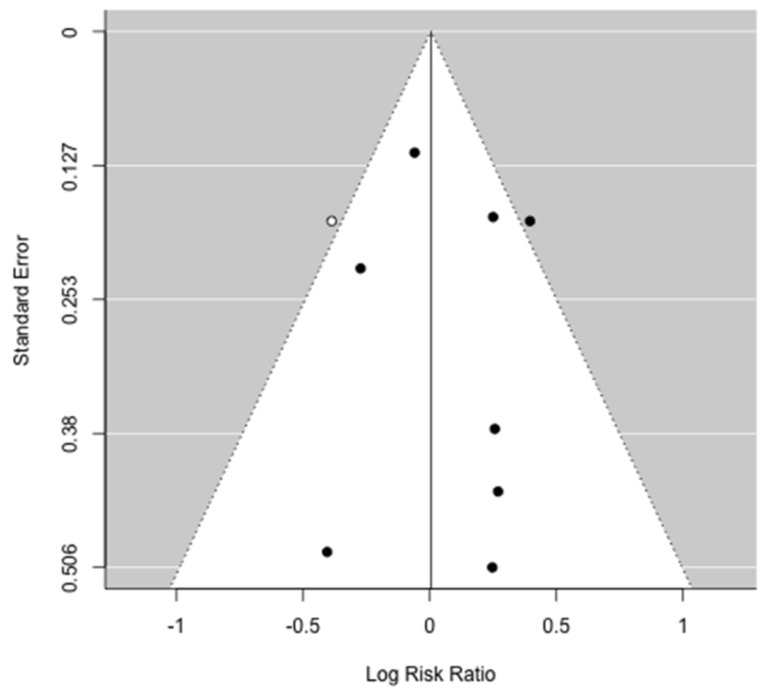
Funnel plot for the prevalence ratio of major complications after pancreatic resection. Black circles identified studies included in the meta-analysis. Publication bias analysis estimated one study missing (white circle).

**Figure 4:**
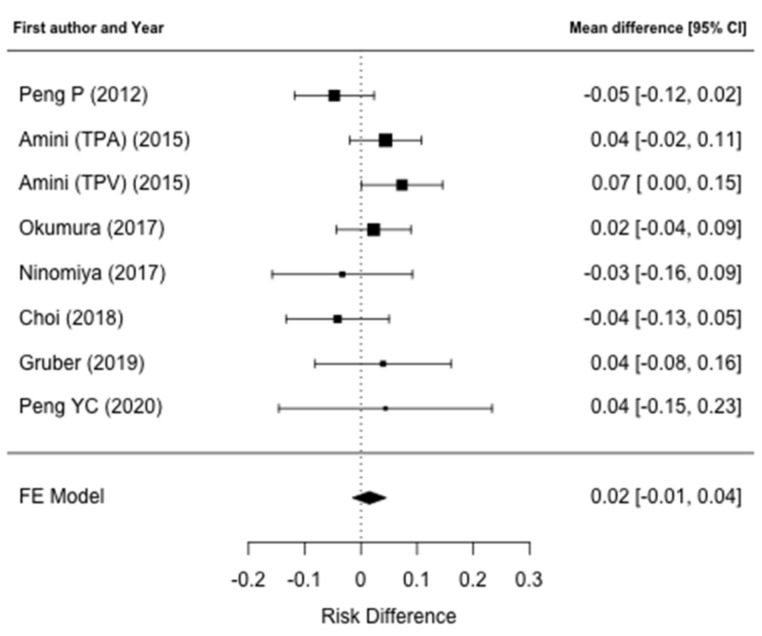
Forest plot for the difference in the prevalence of major complications. The difference in prevalence of major complications in patients with vs. without low muscle mass was not significant.

**Figure 5:**
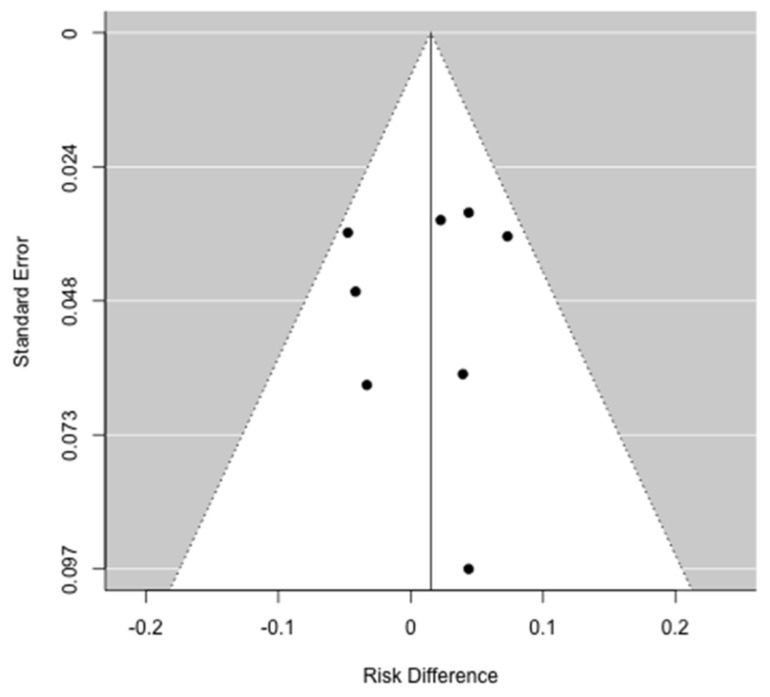
Funnel plot for the difference in the prevalence of major complications. No publication bias was evident.

Data regarding postoperative outcomes were reported in nine studies [31,32,34,35,36,37,38,39,40]. The comparison of overall morbidity rates between patients who have low muscle mass and non-low muscle mass were reported in seven papers [31,32,35,36,38,39,40]. An increased postoperative morbidity rate in low muscle mass patients was found only by Amini et al. [32] and patients with a lower TPV were at a higher risk for postoperative complications (OR: 1.79, 95% CI: 1.25–2.56; *p* = 0.002). Moreover, in a multivariate logistic regression model, TVP-sarcopenia was confirmed to be independently associated with a higher risk for postoperative complications (OR: 1.69, 95% CI: 1.16–2.46; *p* = 0.006). Regarding specific postoperative complications, two papers [35,39] reported the rate of pancreatic fistula between the sarcopenic and non-sarcopenic group, although no correlation was found with low muscle mass. Data on 90-day postoperative mortality were reported in four papers [31,32,35,36] and no differences were noted in regard to muscle mass status. Complete data on major postoperative complications and on postoperative LOS were reported by seven [31,32,35,36,38,39,40] and four papers [31,32,38,40], respectively, and were included in the meta-analysis. Meta-analysis failed to identify a higher prevalence ratio of major complications after pancreatic surgery in the low muscle mass group (PR: 1.07; 95% CI: 0.93–1.24, *p* = 0.22) (Figure 2). There was no heterogeneity between studies (I^2^ = 0%, *p* = 0.70) and publication bias analysis estimated one study missing, nonetheless obtaining comparable results (PR: 1.00; 95% CI: 0.88–1.15, *p* = 0.95) (Figure 3). The difference in the prevalence of major complications in patients with vs. without low muscle mass was 0.02 (95% CI: −0.01–0.04, *p* = 0.32) (Figure 4). There was some heterogeneity between studies (I^2^= 18.8%, *p* = 0.28). There was no evidence of publication bias (Figure 5).

Meta-analysis failed to identify an increase in the mean LOS of patients with or without low muscle mass (ROM: 1.08; 95% CI: 0.97–1.20, *p* = 0.17). There was heterogeneity between the studies (I^2^ = 64.3%, *p* = 0.02) without any publication bias. Similarly, the difference of the mean LOS was not significantly different between the two groups (low muscle mass vs. non-low muscle mass) (MD: 0.8; 95% CI: −0.3–1.9, *p* = 0.14). There was heterogeneity (I^2^ = 52.6%, *p* = 0.076) and no publication bias was present.

Moreover, some authors investigated the correlation between postoperative outcomes and sarcopenic obesity or muscle attenuation. Amini et al. reported that patients with sarcopenic obesity based on TPV had a more pronounced risk of complications compared with patients who did not have sarcopenia (TPV-sarcopenic obesity, 74.1% vs. non-sarcopenia 42.2%, *p* = 0.003) [32]. Peng YC et al. found no significant differences between sarcopenic patients and sarcopenic obese patients in terms of LOS and major complications [40]. Okumura compared patients with or without sarcopenic obesity and found no correlation in terms of major complications or postoperative pancreatic fistula incidence [35]. Furthermore, Okumura investigated the correlation between muscle attenuation and the postoperative outcomes, finding no correlation between myosteatosis and major complications or pancreatic fistula [35]. Apart from the study of Okumura et al. [35], Choi et al. also found no correlation between low muscle attenuation and the overall morbidity rate [38].

### 3.5. Low Muscle Mass and Survival

**Figure 6:**
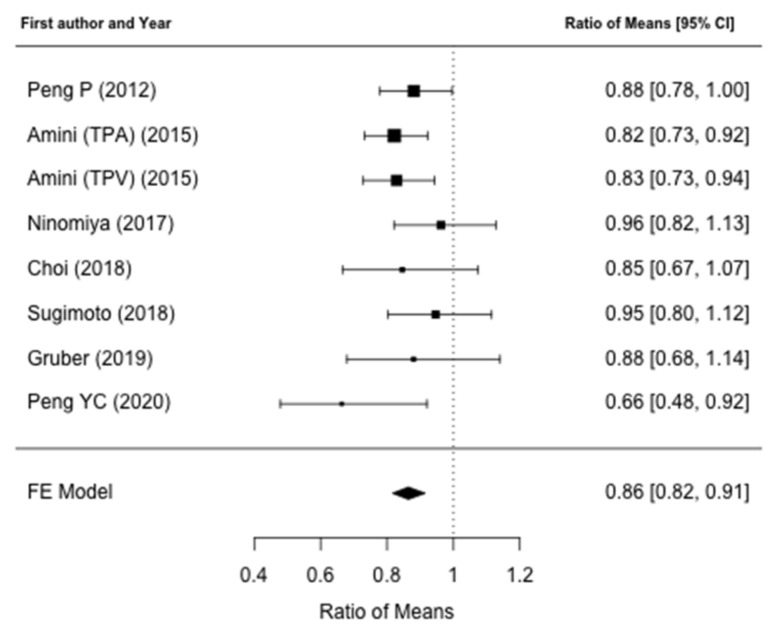
Forest plot for the difference ratio of overall survival. Meta-analysis identified that patients with low muscle mass who underwent pancreatic resection demonstrated a significantly reduced OS when compared to patients without alterations of the muscle mass.

**Figure 7:**
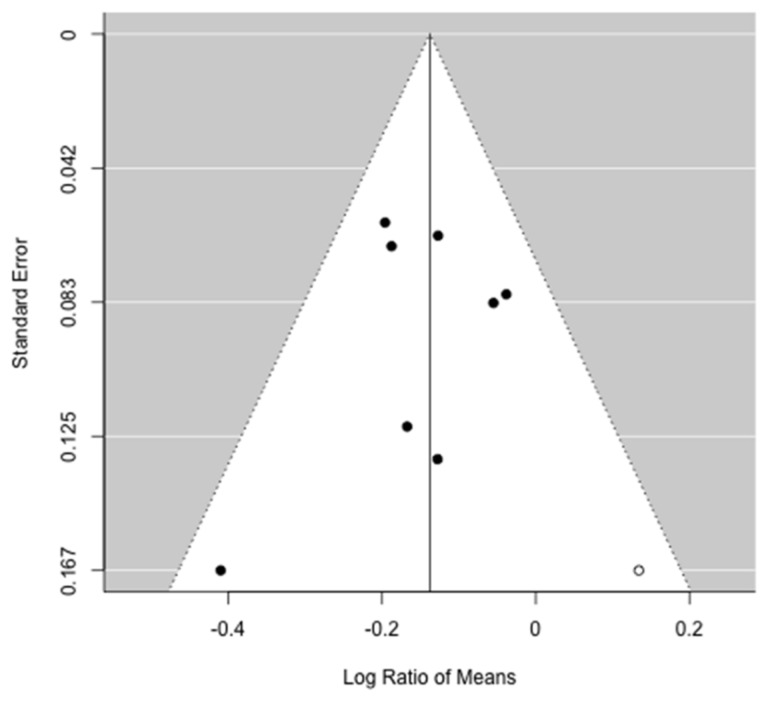
Funnel plot for the difference ratio of overall survival. Publication bias analysis estimated one study missing (white circle).

**Figure 8:**
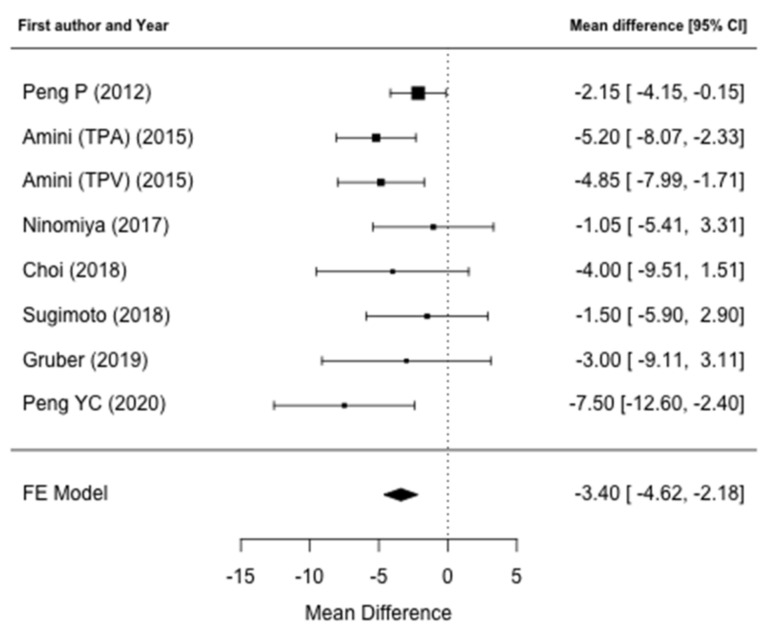
Forrest plot for the mean difference of overall survival. The mean survival loss for patients with low muscle mass was 3.4 months.

**Figure 9:**
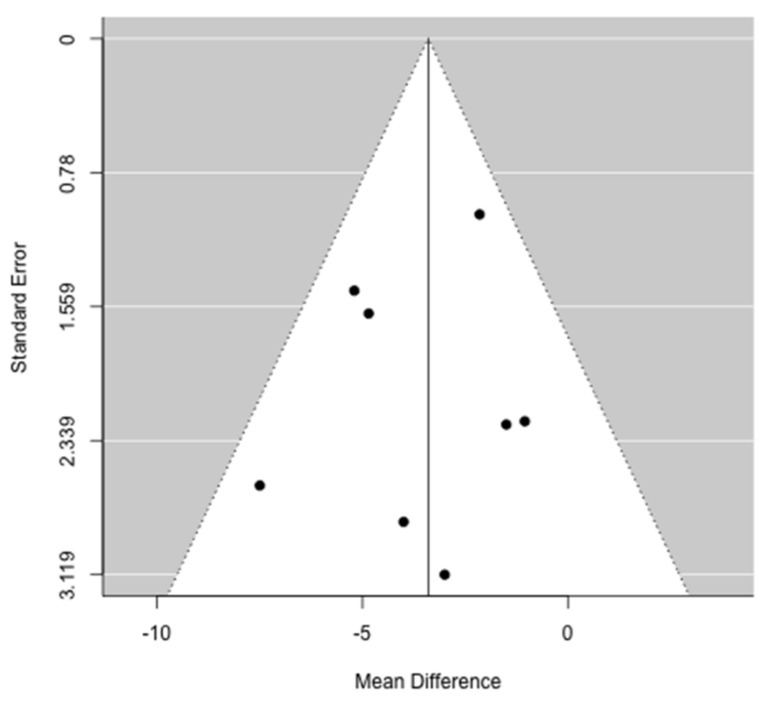
Funnel plot for the mean difference of overall survival. No publication bias was identified.

The effects of alterations of preoperative muscle mass on OS were reported in nine studies [31,32,34,35,36,37,38,39,40]. Seven studies were included in the meta-analysis [31,32,36,37,38,39,40], in which two studies’ [34,35] data on survival required for meta-analysis could not be retrieved in the text. Meta-analysis identified that patients with low muscle mass who underwent pancreatic resection demonstrated a significantly reduced OS when compared to patients without alterations of the muscle mass (ROM: 0.86; 95% CI: 0.82–0.91, *p* < 0.001), resulting in a 14% loss for the former (Figure 6). There was no heterogeneity between studies (I^2^ = 0%, *p* = 0.46) and publication bias analysis estimated one study missing, nonetheless obtaining comparable results (ROM: 0.87; 95% CI: 0.82–0.92, *p* < 0.001) (Figure 7). The mean survival loss for patients with low muscle mass was 3.4 months (95% CI: −4.62, −2.18 *p* < 0.001) (Figure 8). There was some heterogeneity between studies (I^2^ = 14.6%, *p* = 0.32) with no publication bias identified (Figure 9). Nine studies performed multivariate analysis, identifying low muscle mass as a significant independent risk factor for mortality [31,32,34,35,36,37,38,39,40].

Moreover, five studies [35,37,38,39,40] analyzed the impact of low muscle mass on the DFS. Okumura determined that DFS rates were significantly lower in patients with low muscle mass [35] and Sugimoto et al. reported that a smaller sex-standardized SMI was independently associated with a shorter DFS [37]. On the contrary, three studies found that DFS was not significantly different between patients with or without sarcopenia [38,39,40]. As data were missing, meta-analysis was not possible. Regarding sarcopenic obesity, three authors [35,39,40] reported data regarding the OS and DFS. Peng YC et al. [40] found an association in the univariate analysis between sarcopenic obesity and OS (HR = 3.19, 95% CI = 0.98–10.37, *p* = 0.041), although data were not confirmed in the multivariate analysis (HR = 1.29, 95% CI = 0.23–7.19, *p* = 0.768). Okumura et al. [35] found a correlation between sarcopenic obesity and OS both in the univariate (HR = 1.91, 95% CI = 1.30–2.75, *p* = 0.001) and multivariate analysis (HR = 2.01, 95% CI = 1.31–3.03, *p* = 0.002). Gruber et al. reported an impaired OS in the obese sarcopenic patients compared to non-sarcopenic obese [39]. While Peng YC et al. [40] and Gruber at al. [39] found no association between sarcopenic obesity and DFS, Okumura et al. [35] found the association to be relevant both in the univariate (HR = 1.83, 95% CI = 1.31–2.53, *p* = 0.001) and multivariate analysis (HR = 1.87, 95% CI = 1.32–2.61, *p* = 0.001). Two authors [38,40] found no association between muscle attenuation and OS or DFS. On the contrary, Okumura et al. [35] found a significantly reduced OS and DFS in patients with preoperative reduced muscle attenuation both in the univariate (HR = 1.93, 95% CI = 1.40–2.67, *p* < 0.001 for OS; HR = 1.56, 95% CI = 1.18–2.07, *p* = 0.002 for DFS) and multivariate analysis (HR = 1.63, 95% CI = 1.13–2.36, *p* = 0.01 for OS; HR = 1.37, 95% CI = 1.02–1.84, *p* = 0.037 for DFS).

## 4. Discussion

Cancer cachexia is defined as a multifactorial syndrome characterized by ongoing loss of skeletal muscle mass (with or without loss of fat mass) that can be partially but not entirely reversed by conventional nutritional support [6]. This muscle loss is defined as secondary sarcopenia, also known as disease-related sarcopenia, in which a causal factor other than (or in addition to) aging is evident [5]. As opposed to primary sarcopenia, secondary sarcopenia has predominantly focused on the loss of muscle mass without an emphasis on muscle function [48]. Indeed, none of the retrospective studies considered in this review documented muscle strength or performance. Secondary sarcopenia could represent an individual characteristic to target in order to improve the outcome. In fact, patients with solid tumors frequently experience malnutrition due to reduced food intake, malabsorption, energy expenditure, and altered metabolism. Treatment options include physical training, modifications of nutritional intake (including appetite stimulants), and pharmacological treatment tested in clinical trials [49]. Among solid tumors, pancreatic cancer carries the highest prevalence of cancer cachexia and weight loss [49]. Its overall survival rate is still dismal with little improvements over the last decade [50] and postoperative complications remain an important burden after pancreatic surgery, with morbidity rates still up to 40% [18]. Surgical complications such as pancreatic fistula, hemorrhage, and delayed gastric emptying not only affect patient convalescence and quality of life but negatively impact oncological outcomes, delay adjuvant treatment, and affect survival [51]. Sarcopenia has been proposed as an indicator of frailty and therefore as a potential mean to predict the risk of postoperative morbidity [52]. In fact, low muscle mass or radiodensity can lead to impaired wound healing, depressed immunity, and inability to mobilize after surgery, thus affecting postoperative outcomes [53]. While several studies have reported the association between sarcopenia and outcomes following surgery for various oncologic diseases [54], the actual impact of sarcopenia on surgical morbidity after pancreatic surgery and on survival remains poorly defined with a high heterogeneity of results. As depicted by our meta-analysis, sarcopenia plays a significant role in the OS, while the influence on postoperative outcomes remains uncertain. The meta-analyses we conducted failed to demonstrate a certain relationship between low muscle mass and major complications or LOS. On the contrary, other authors have found a correlation between low muscle mass and postoperative outcomes [55]. The inhomogeneity among the considered populations could be a possible explanation of the different results reported. Another potential bias to be considered is the different assessment parameters used to define the presence of low muscle mass. Similarly to Amini et al. [32], previous studies reported divergent results when using different assessment parameters. In addition, Pecorelli et al. [9] reported that sarcopenia using the total abdominal muscle area (TAMA) was not a significant prognostic factor for 60-day postoperative mortality (*p* = 0.224). However, the ratio of visceral fat area (VFA) to TAMA was found to be a significant predictor for 60-day mortality when the ratio was 3.2 in the multivariate analysis [OR 6.76, 95% CI: 2.42–18.99; *p* < 0.001]. The lack of a univocal definition of sarcopenia and, even worse, too many different self-determined cut-offs, obtained by means of optimum stratification in populations with different ethnicities, BMI results, age, and cancer types, determine a void in research and clinical practice. For instance, it is worth noticing that cut-offs from previous western studies, such as in Prado et al. [26], might be inappropriate for Asian populations such as that studied by Ninomiya et al [36]. Moreover, the cut-offs described by Prado et al. were obtained in a subset of obese patients (BMI > 30) and therefore their application on non-obese patients may be inappropriate.

In fact, the study of sarcopenia in humans is complicated by the large variability among individual and multiple factors affecting muscle (comorbidities, drugs, lifestyle, nutritional aspects, and environmental influences), which can vary in different populations. This muscle loss (secondary sarcopenia) is caused or worsened by cancer treatments and the tumor itself. Moreover, different studies are focused on different muscles and presently there is no consensus in the methodology of the assessment of muscle mass in the diagnosis of sarcopenia or cancer cachexia. Despite the importance of evaluating muscle mass in cancer, the definition of “low” muscle mass is difficult to be standardized when different cut-off values are applied. As depicted in our literature review, all included studies used a different cut-off to define sarcopenia and the reported prevalence of low muscle mass varied from 17.2% to 64.2%. Hence, more collective and coordinated efforts are required to compile and compare data obtained in different populations of cancer patients.

The rising subject in the field of muscle wasting and frailty regards the quality of the muscle rather than the quantity. Akahori et al. [56] focused on the muscle density as a possible prognostic factor in pancreatic patients and found a significant association between reduced muscle attenuation after chemo-radiotherapy and overall survival. Similarly, other authors found a correlation of a progression/outcome of cancer with muscle attenuation [7,15,27,53,57]. Moreover, some recent results demonstrated that sarcopenia and myosteatosis represent two separate and distinct clinical phenotypes accompanied by different biological profiles in patients with pancreatic adenocarcinomas [53]. Yet again, there are no standardized cut-offs and thus it is difficult to compare the literature results.

Our study has some limitations to consider. The relatively small number of studies analyzed and their heterogeneity and retrospective nature could represent a significant risk of selection bias. Moreover, due to the lack of data in some studies, we could not measure outcomes such as overall postoperative morbidity rates or specific complications of pancreatic surgery. Therefore, we were unable to fully investigate the potential role of low muscle mass on postoperative short-term outcomes. New prospective and multicentric studies are necessary in order to draw more definitive results.

## 5. Conclusions

Although we cannot draw unequivocal conclusions, we can expect sarcopenia to have an impact on the surgical and oncological outcomes of cancer patients. Our meta-analysis on patients with PDAC undergoing surgery demonstrates a reduced survival in those with sarcopenia; however, a clear correlation with the short-term postoperative outcomes was not evident. We believe results can be compromised by the diverse definitions and cut-off values utilized. We advocate a joint effort to standardize body composition evaluation methods, assessment parameters, and cut-off values. This enables risk stratification in order to implement nutritional and pre-/re-habilitation interventions with the aim of reducing physical disability, improving the quality of life, and prolonging survival.

## Figures and Tables

**Table 1:** Terminology and definitions of sarcopenia in the included studies.

Author	Measurements of Skeletal Muscle	Criteria to Define Sarcopenia	Cut-Off Values Males	Cut-Off Values Females	Definition of Sarcopenic Obesity	Definition of Myosteatosis
Peng P et al. [31]	TPA (L3)	Quartiles	Lowest quartile: 492 mm^2^/m^2^	Lowest quartile: 362 mm^2^/m^2^	Sarcopenia + BMI ≥ 30 kg/m^2^	-
Amini et al. [32]	TPA (L3) and TPV (L3)	Quartiles	Lowest quartile TPA: 564.2 mm^2^/m^2^ TPV: 17.2 cm^3^/m^2^	Lowest quartile TPA: 414.5 mm^2^/m^2^; TPV: 12.0 cm^3^/m^2^	Sarcopenia + BMI ≥ 30 kg/m^2^	-
Clark et al. [33]	CSAPM/CSAL5	Linear regression analysis with survival	-	-	-	-
Delitto et al. [34]	CSAPM/CSAL3	Linear regression analysis and Median	CSAPM/CSAL3 < 0.58	CSAPM/CSAL3 < 0.58	-	-
Okumura et al. [35]	SMI	Self-determined cut-offs (in relation to 3-year mortality)	47.1 cm^2^/m^2^	36.6 cm^2^/m^2^	Low SMI + VFA ≥ 100 cm^2^	<35.1 HU (Male) <30.7 HU (Female)
Ninomiya et al. [36]	SMI	Prado 2008 [26] (only for females)	43.75 cm^2^/m^2^	38.5 cm^2^/m^2^	Sarcopenia + BMI ≥ 22 kg/m^2^	-
Sugimoto et al. [37]	SMI	Quartiles	Lowest quartile	Lowest quartile	-	-
Choi et al. [38]	SMI	Tertiles	Lowest tertile 45.3 cm^2^/m^2^	Lowest tertile 39.3 cm^2^/m^2^	-	<40.8 HU (Male) <33.9 HU (Female)
Gruber et al. [39]	SMI	Prado 2008 [26]	52.4 cm^2^/m^2^	38.5 cm^2^/m^2^	Sarcopenia + BMI ≥ 25 kg/m^2^	-
Peng YC et al. [40]	SMI	Choi 2015 [41]	42.2 cm^2^/m^2^	33.9 cm^2^/m^2^	Sarcopenia +VAT/TAMA ≥ 2	<41 HU with BMI < 25 kg/m^2^ <33 HU with BMI ≥ 25 kg/m^2^

TPA (L3): total psoas area measured at the level of L3 normalized for the square of the height; TPV (L3): total psoas volume measured at the level of L3 normalized for the square of the height. A total of 55 cm of the total psoas length was assessed in Amini et. al.; CSAPM/CSAL5: cross-sectional area of the psoas muscle at the L5 vertebral level standardized to the L5 cross-sectional area of the body (CSAL5); CSAPM/CSAL3: cross-sectional area of the psoas muscle at the L3 vertebral level standardized to the L3 cross-sectional area of the body (CSAL3); SMI (skeletal muscle index): cross-sectional area of the muscle at the L3 level normalized for the square of the height; VFA: visceral fat area; VAT/TAMA: visceral adipose tissue area/total abdominal muscle area at the L3 vertebral level.

**Table 2:** Characteristics of the studies included in the systematic review.

Author	Year	Country	Study Accrual Period	Study Type	Patients (*n*)
Peng P et al. [31]	2012	Baltimore, USA	1999–2010	RCS	557
Amini et al. [32]	2015	Baltimore, USA	1996–2014	RCS	763
Clark et al. [33]	2016	Tampa, USA	2004–2012	RCS	100
Delitto et al. [34]	2016	Gainesville, FL, USA	2010–2014	RCS	73
Okumura et al. [35]	2017	Kyoto, Japan	2004–2015	RCS	301
Ninomiya et al. [36]	2017	Nagoya, Japan	2005–2014	RCS	265
Sugimoto et al. [37]	2018	Rochester, MN, USA	2000–2015	RCS	323
Choi MH et al. [38]	2018	Seoul, Korea	2008–2015	RCS	180
Gruber et al. [39]	2019	Vienna, Austria	2005–2010	RCS	133
Peng YC et al. [40]	2020	Taipei, Taiwan	2005–2018	RCS	116

RCS = retrospective cohort study.

**Table 3:** Studies included in the quantitative analyses and outcomes used for the three meta-analyses.

Author	Low Muscle Mass	N pts	OS Mo (Range)	*p*	Major Complications *n* (%)	*p*	LOS Days (Range)	*p*
Peng P et al. [31]	Yes	139	13.7	0.01	21 (15.1)	NS	12	0.980
	No	418	18		83 (19.9)		12	
Amini et al. (TPA) [32]	Yes	192	18.0	<0.001	38 (19.8)	0.16	9 (7–15)	0.05
	No	571	28.4		88 (15.4)		8 (7–13)	
Amini et al. (TPV) [32]	Yes	152	17.0	<0.001	34 (22.4)	0.03	10 (7–15.5)	0.002
	No	611	26.7		92 (15.1)		8 (7–13)	
Clark et al. [33]	Yes	NA	NR		NR		NR	
	No	NA	NR		NR		NR	
Delitto et al. [34]	Yes	NA	NA	0.001	NR		NR	
	No	NA	NA		NR		NR	
Okumura et al. [35]	Yes	120	NA	<0.001	12 (10)	0.493	NR	
	No	181	NA		14 (7.7)		NR	
Ninomiya et al. [36]	Yes	170	23.7	0.185	91 (53.5)	0.541	NR	
	No	95	25.8		54 (56.8)		NR	
Sugimoto et al. [37]	Yes	80	23	0.075	NR		NR	
	No	243	26		NR		NR	
Choi et al. [38]	Yes	60	13.9	0.031	5 (8.3)	0.402	15.6 ±7.9	0.303
	No	120	21.9		15 (12.5)		17.2 ±10.8	
Gruber et al. [39]	Yes	78	14 (11–17)	0.016	13 (16.7)	0.531	14	0.243
	No	55	20 (14–26)		7 (12.7)		11	
Peng YC et al. [40]	Yes	20	11.6	0.009	4 (20)	0.630	32 ±22.5	0.51
	No	96	26.6		15 (15.6)		27.6 ±27.5	

OS: overall survival; Mo: months; LOS: length of hospital stay; NA: not available; NR: not reported; and NS: non-significant *p* value.

## Data Availability

The data presented in this study are available within the article.
